# The quality of life of patients with spinal cord injuries

**Published:** 2012-11

**Authors:** Hanieh Shahandeh, Sahar Moradi, Katayoon Bavandpour, Fariba Bohlooli

**Affiliations:** ^*a*^Vice Chancellery for Research and Technology ,Kermanshah University of Medical Sciences, Kermanshah, Iran.

## Abstract

**Background:**

Nowadays, the quality of life is considered among important and applied topics in various human studies, especially in the case of disabled groups.

The objective of the present study is to investigate the quality of life in disabled people with spinal cord injuries who were member of Spinal Cord Injuries (SCI) institute of Tehran.

**Methods:**

For this purpose, 86 persons of SCI members were selected by simple random sampling method. Data were collected by a self-administered questionnaire. The questions emphasized on the self-perception of individuals on the quality of life.

The questionnaire used in this study was adapted from Lancashire and Wisconsin questionnaires in which the social-cultural aspects of Iranian people have been considered. The questionnaire consisted of two parts. The first part included 5 questions on demographic data including age, gender, lesion characteristics such as type and duration of injury. The second part consisted on questions related to the 11-fold dimensions of the quality of life (including employment, education, recreation, economic status, housing, daily living activities, family relationships, social relationships, physical health, mental well-being, self-concept, independence the environment).

**Results:**

The results of the present study showed that 73.3% of patients with spinal cord injury assessed their quality of life as moderate and good with almost same percentage. The examination of 11-fold dimensions of the quality of life showed that the mean score of the quality of life of the studied group in the social relations was higher than the score obtained in the other dimensions (3.67 of 5 points). However, in the case of married individuals, the mean score of the quality of life in the family relationship was higher than this value (3.95 of 5 points).

The examination of the relationship between the quality of life and age, gender, cause, type and duration of the lesion showed that the quality of life in women with spinal cord injury was superior than men (p less than 0.01). The mean score of the quality of life for women and men was 3.3 and 2.9 of 5 points, respectively. However, no significant relationship was found between the quality of life and age, cause of injury, type of injury and duration of injury.

**Conclusions:**

The results indicate this fact that a high percentage of cases expressed the level of their life quality as moderate and good which is very close to the quality of life reported in individuals with spinal cord injury in other parts of the world.

**Keywords:**

Quality of life, Spinal cord injury, Disable groups, Self administered questionnaire


Volume 4, Suppl. 1 Nov 2012
Publisher: Kermanshah University of Medical Sciences
URL: http://www.jivresearch.org
Copyright: (cc) BY

**Paper No. 73**

### The quality of life of patients with spinal cord injuries

Hanieh Shahandeh [a,*], Sahar Moradi [a], Katayoon Bavandpour [a], Fariba Bohlooli [a]

[a] Vice Chancellery for Research and Technology, Kermanshah University of Medical Sciences, Kermanshah, Iran.

### Abstract:

**Bckground:** Nowadays, the quality of life is considered among important and applied topics in various human studies, especially in the case of disabled groups.
The objective of the present study is to investigate the quality of life in disabled people with spinal cord injuries who were member of Spinal Cord Injuries (SCI) institute of Tehran.
**Methods:** For this purpose, 86 persons of SCI members were selected by simple random sampling method. Data were collected by a self-administered questionnaire. The questions emphasized on the self-perception of individuals on the quality of life.
The questionnaire used in this study was adapted from Lancashire and Wisconsin questionnaires in which the social-cultural aspects of Iranian people have been considered. The questionnaire consisted of two parts. The first part included 5 questions on demographic data including age, gender, lesion characteristics such as type and duration of injury. The second part consisted on questions related to the 11-fold dimensions of the quality of life (including employment, education, recreation, economic status, housing, daily living activities, family relationships, social relationships, physical health, mental well-being, self-concept, independence the environment).
**Results:** The results of the present study showed that 73.3% of patients with spinal cord injury assessed their quality of life as moderate and good with almost same percentage. The examination of 11-fold dimensions of the quality of life showed that the mean score of the quality of life of the studied group in the social relations was higher than the score obtained in the other dimensions (3.67 of 5 points). However, in the case of married individuals, the mean score of the quality of life in the family relationship was higher than this value (3.95 of 5 points).
The examination of the relationship between the quality of life and age, gender, cause, type and duration of the lesion showed that the quality of life in women with spinal cord injury was superior than men (p less than 0.01). The mean score of the quality of life for women and men was 3.3 and 2.9 of 5 points, respectively. However, no significant relationship was found between the quality of life and age, cause of injury, type of injury and duration of injury.
**Conclusions:** The results indicate this fact that a high percentage of cases expressed the level of their life quality as moderate and good which is very close to the quality of life reported in individuals with spinal cord injury in other parts of the world.

### Keywords:

***Quality of life, Spinal cord injury, Disable groups, Self administered questionnaire***

*** Corresponding Author at:**
***Hanieh Shahandeh:*** *Vice Chancellery for Research and Technology, Kermanshah University of Medical Sciences, Kermanshah, Iran. Tel: 0831-8393159, Email: hanieh1384@yahoo.com,* ***(Shahandeh H.).***

